# Novel approaches to HIV therapy

**DOI:** 10.12688/f1000research.11164.1

**Published:** 2017-05-31

**Authors:** Eric S. Daar

**Affiliations:** 1 Department of Medicine, Harbor-UCLA Medical Center, Torrance, CA 90502, USA

**Keywords:** HIV therapy, NRTI, NNRTI

## Abstract

There are approximately 35 million people infected by human immunodeficiency virus (HIV), with an estimated 2 million incident infections annually across the globe. While HIV infection was initially associated with high rates of morbidity and mortality, advances in therapy have transformed it into a chronic and manageable disease. In addition, there is very strong evidence that those on antiretroviral therapy are much less likely to transmit infection to their partners. The success rates for maintaining viral suppression in treated patients has dramatically increased owing to the development of agents that are potent and well tolerated and can often be co-formulated into single pills for simplification. This review will outline advances in treatment over the last several years as well as new strategies that may shift the existing treatment paradigm in the near future.

## Introduction

The development of potent antiretroviral therapy (ART) for the treatment of human immunodeficiency virus (HIV) infection has been one of the greatest advances in modern medicine. While therapy has become increasingly easy to take and well tolerated, there remain limitations that prevent some patients from benefiting from treatment. Future goals of therapy are to overcome the limitations of current treatment, including side effects and the need to take medication on a daily basis.

Early versions of potent ART that were able to suppress HIV to undetectable levels consist of regimens that included two nucleoside reverse transcriptase inhibitors (NRTIs) plus either a protease inhibitor (PI) or a non-NRTI (NNRTI), often requiring relatively frequent dosing, had substantial side effects, frequent drug-drug and drug-food interactions, and at times were associated with a high risk of virologic failure and emergent drug-resistant virus. The next few decades of drug development focused on overcoming these limitations of treatment. Initial success included the use of pharmacologically boosted PIs that were easier to take, had fewer drug-food interactions, and were rarely associated with the emergence of drug-resistant virus. In addition, dosing frequency was simplified, ultimately to once-daily regimens. Simultaneously, there was the emergence of new NRTIs that were given as fixed-dose combinations once daily and associated with less toxicity. The next major advance in treatment was the development of a new class of therapy, integrase strand transfer inhibitors (INSTIs). Overriding all of these advances was the emergence of increasing numbers of fixed-dose combinations, including regimens that could be given as a single pill once per day. This review will summarize the advances in the last several years that have revolutionized ART along with new drugs and novel treatment strategies on the horizon.

## Current options for first line antiretroviral therapy

The evolution of new antiretroviral agents resulted in the availability of the first truly potent regimens in 1996 that were able to suppress plasma HIV RNA to undetectable levels and prevent the emergence of drug-resistant virus. Early regimens included two NRTIs with either non-pharmacologically boosted PIs or NNRTIs. While these regimens saved lives, they were difficult to take, had numerous side effects, and, when failure occurred, were often associated with the emergence of drug-resistant virus. These limitations of therapy resulted in most guidelines suggesting that treatment should be deferred until patients were at high risk for the development of acquired immunodeficiency syndrome (AIDS)-associated complications, such as when CD4 cell counts were less than 200 cells/µL. Fast forward over the ensuing 10 years and drug development focused on agents which were easier to take, less likely to be associated with treatment failure, and often active against viruses that had underlying drug resistance. This included the recognition that pharmacologically boosted PIs could be given less frequently and were rarely associated with the emergence of drug-resistant virus, a novel observation that represented a major advance in treatment. Nevertheless, there remained substantial side effects and the need for multiple pills, and the pharmacologic-boosting agents were associated with many drug-drug interactions. These early advances did make therapy more palatable and allowed for the consideration of earlier initiation of treatment. In fact, therapy is now recommended for all HIV-infected individuals regardless of stage of disease ^1, 2^. This is largely because of improvements in treatment options as well as data showing that treated individuals are at very low risk of transmitting to partners and progressing to AIDS and non-AIDS-related events ^3, 4^.

If we now advance to the last several years, the field is marked by the emergence of NRTIs that can be given as fixed-dose combinations with far less toxicity than earlier agents in the class. In particular, this represented a move away from zidovudine, didanosine, and stavudine to abacavir (ABC) and tenofovir disoproxil fumarate (TDF). The latter drugs were co-formulated as once-daily regimens with lamivudine (3TC) and emtricitabine (FTC), respectively. While the availability of these agents represented major advances, including TDF/FTC being co-formulated with the NNRTI efavirenz (EFV) for the first single-tablet regimen, they were not without limitations. ABC has been associated in some studies with increased risk of cardiovascular events ^5^, and TDF is related to nephrotoxicity and more rapid declines in bone mineral density than is seen with other drugs ^6, 7^. In addition, the NRTIs were always combined with a “third” drug, usually a pharmacologically boosted PI, NNRTI, or more recently an INSTI, the newest class of antiretrovirals. In fact, treatment guidelines since the introduction of potent ART more than 20 years ago have continued to recommend regimens that include two NRTIs plus a third drug as preferred options ^1, 2^. In light of the many excellent treatment options, therapy is individualized based upon patient preference and the presence or absence of transmitted resistance and comorbid conditions, such as renal, bone, and cardiovascular problems as well as co-infections such as viral hepatitis and tuberculosis. Finally, although data are limited, ongoing research is attempting to better characterize how different treatments might impact the elevated levels of systemic inflammation and immune activation seen in virologically suppressed individuals that might adversely affect clinical outcomes ^8, 9^.

In considering the advances over the last several years and on the horizon, it is worth focusing on those related to emerging NRTIs, “third” drugs combined with NRTIs, and the possibility of treating people with regimens that do not include the traditional two NRTIs plus a third drug.

## Evolution of nucleoside reverse transcriptase inhibitors in first-line therapy

For the majority of patients, there have been few problems associated with regimens including ABC/3TC or TDF/FTC. Nevertheless, the observations in select studies that the use of ABC was associated with increased risk of cardiovascular events, especially in those with multiple other cardiovascular risk factors, did pose some concerns for select patients ^5^. The relationship between TDF and renal disease and declining bone mineral density was similarly problematic for those with underlying renal disease or other comorbid conditions, such as diabetes mellitus, hypertension, and osteopenia/osteoporosis ^6, 7^. In fact, as patients living with HIV get older, clinicians are frequently encountering those with multiple comorbid conditions including underlying renal disease and cardiovascular risk factors. A challenge in this setting was how to select between NRTI-based regimens. In this setting, clinicians either made the best decision possible with close monitoring or needed to consider new options which included regimens that did not include ABC or TDF, or more recently the use of an alternative formulation of tenofovir, tenofovir alafenamide (TAF).

The development of TAF, along with the approval of multiple co-formulations that include this as an alternative to TDF, represents an important advance in treatment (reviewed in 10). The rationale for using TAF instead of TDF, both of which are prodrugs of tenofovir, is that the former can be given as a smaller dose and therefore reduced pill size for co-formulations and is concentrated in lymphoid tissue, where the drug works with much lower levels in plasma with the hope of fewer off-target side effects. In fact, one head-to-head comparison of a regimen with TDF versus TAF and several switch studies where patients changed from TDF- to TAF-based regimens all showed that the latter was associated with equivalent efficacy, probably less nephrotoxicity, and clearly less of an effect on bone mineral density. TAF is now approved for use in anyone with estimated creatinine clearance of at least 30 mL/minute and is available co-formulated with commonly used regimens including FTC/TAF, rilpivirine (RPV)/FTC/TAF, and elvitegravir (EVG)/cobicistat (COBI)/FTC/TAF. Many guidelines have embraced this new formulation of tenofovir, with the International Antiviral Society-USA recommending that this be the preferred tenofovir-based regimen unless not available ^2^ and the Department of Health and Human Services guidelines considering it an equivalent option to TDF ^1^. A few important caveats are noted with regard to TAF, including that the data in people with advanced renal disease, i.e. estimated creatinine clearance <50 mL/minute, are limited. In addition, TAF cannot be used with rifamycins, there are limited data and therefore it is not yet recommended in pregnancy, and there are no data using it for pre-exposure prophylaxis and therefore it is not an option in this setting. Although there are some new NRTIs in early stages of clinical development, the next big step for this class of drugs is the emergence of treatment strategies that may not include them at all, or at least not ABC, TDF, or TAF.

## Evolution of third drugs to be combined with nucleoside reverse transcriptase inhibitors in first-line therapy

Treatment guidelines over the past several years have continued to focus on pharmacologically boosted PIs, NNRTIs, and more recently INSTI-based options ^1, 2^. In the arena of pharmacologically boosted PIs, it has become clear that once-daily atazanavir (ATV) and darunavir (DRV) boosted by either ritonavir (RTV) or COBI are the best-tolerated options with high levels of efficacy. In addition, this class of drugs has long been known to have the unique property of rarely selecting for drug resistance, even in those experiencing virologic rebound. Recent data from a head-to-head comparison showed that DRV was as effective as ATV but somewhat better tolerated, mostly because of increased hyperbilirubinemia known to occur with ATV, a benign and reversible side effect of therapy ^11^. That said, with few advantages of ATV over DRV, most guidelines have favored the latter, with a particular role in those where there are concerns regarding poor adherence or when treatment needs to be started before baseline drug-resistance testing is available ^1^. The availability of fixed-dose combination of both PIs with COBI further simplifies these regimens in clinical practice ^12, 13^. The next potential big advance for this class relates to an ongoing study of a single-tablet regimen of DRV/COBI/FTC/TAF. Assuming data from phase III trials of this regimen are consistent with what has been seen in phase II studies ^14^, this would be the first PI regimen that could be taken as a single pill per day.

The role of NNRTIs as a third drug has been on the decline of late as a result of the expansion of the INSTIs into clinical practice, as described below. While EFV-based regimens had been the mainstay of treatment for many years, especially as the first drug included in a single-tablet regimen, there has been a shift away from this drug owing to well-known central nervous system side effects, and the development of multiple other better-tolerated drugs, including INSTIs. RPV is another NNRTI that is listed as a viable treatment option and comes as part of a single-tablet regimen with FTC and either TDF or TAF. This drug is well tolerated and effective, although potentially less so in those with plasma HIV RNA >100,000 copies/mL and CD4 cell counts <200 cells/µL ^15^. In addition, it requires dosing with a reasonably sized meal and avoidance of acid-reducing agents. Another option on the horizon is doravirine, a new NNRTI that is given once-daily, has few side effects, and appears to be effective regardless of baseline CD4 cell count and plasma HIV RNA ^16^.

The INSTIs are clearly the new darlings of ART. The first in this class was raltegravir (RAL), which has been shown to be highly effective and extremely well tolerated and has a paucity of drug-drug and drug-food interactions ^17^. A limitation has been the need for twice-daily dosing; however, a new formulation has been developed and shown to be as effective and as well tolerated as currently approved twice-daily dosing but will be given as two pills once per day ^18^. The second drug in this class was EVG, which needs to be pharmacologically boosted and is part of EVG/COBI/FTC/TDF and the same three drugs with TAF that has more recently been approved. This regimen has been thoroughly studied and shown to be highly effective with good tolerability ^19, 20^. It is associated with drug-drug interactions and, like RAL, the emergence of INSTI resistance in the select few patients who experience virologic failure. Moreover, there is cross-resistance between these two drugs. Finally, the most recently approved INSTI is dolutegravir (DTG), which does not require boosting, is highly effective, and is generally well tolerated. A unique observation with this drug is that, in four fully-powered phase III randomized controlled trials, there has yet to be anyone who failed with resistance to any drugs in the regimen ^21– 24^. Based upon this, along with other data, it is believed that the barrier to resistance for this INSTI might be higher than that of other drugs in the class. It comes as a single-tablet regimen with ABC/3TC. The lack of resistance seen with this drug has raised the possibility of using it in novel combinations, several of which are discussed in the section on novel regimens below. The next potential advance in this class will be the availability of another INSTI called bictegravir that does not require pharmacologic boosting and will be combined with FTC/TAF as a single-tablet regimen. Recent phase II data show promise for safety and efficacy ^25^, with several large randomized controlled trials underway for both first-line and switch therapy in virologically suppressed patients.

## Novel regimens

Recent studies have attempted to deviate from the long-held paradigm of two NRTIs plus a third drug. This has largely been driven by the desire to reduce toxicity and potentially the cost of standard regimens while taking advantage of the high barrier to resistance of pharmacologically boosted PIs. In fact, there are now two large fully powered trials of first-line therapy with novel combinations that do not include tenofovir or ABC. This includes lopinavir/RTV (LPV/r) plus 3TC, which was as efficacious as LPV/r plus two NRTIs ^26^. Notably, these data use a pharmacologically boosted PI no longer preferred in guidelines owing to inferior tolerability when compared with DRV and ATV. While one could likely extrapolate these data to more commonly used PIs, the data for the latter do not exist. The other large study was with DRV/RTV once-daily plus RAL twice-daily, which was overall as effective as DRV/RTV with two NRTIs ^27^. However, in the subset of patients with plasma HIV RNA >100,000 copies/mL and CD4 cells <200 cells/µL, it was not as effective. These studies demonstrate that there are options that do not include ABC, TDF, or TAF for first-line therapy. Another potential strategy that is in advanced stages of study for first-line therapy is DTG plus 3TC. This option uses two once-daily agents and does not include tenofovir, ABC, or a pharmacologically boosted PI. A small pilot study included 20 people with screening plasma HIV RNA <100,000 copies/mL and CD4 cells >200 cells/µL and demonstrated high levels of efficacy, with 18 out of 20 patients virologically suppressed at 48 weeks ^28^. Of the two not suppressed, one committed suicide during the study, an outcome not thought to be related to the study drug, and the other had low-level viremia that re-suppressed without a change in their regimen. A fully powered phase III study is underway with this regimen in participants with plasma HIV RNA levels of up to 500,000 copies/mL.

Other novel regimens are being pursued but for “maintenance therapy” in those already suppressed on standard therapy. There are now several large studies looking at this strategy in those with pharmacologically boosted PIs as monotherapy, which worked well but in general demonstrated a somewhat higher rate of virologic failure than did standard therapy ^29^. Notably, in these studies, those with virologic failure rarely had resistance, and when NRTIs were added to the regimen they did suppress plasma HIV RNA. There are also studies of those without any underlying drug resistance who were stable on two NRTIs plus a pharmacologically boosted PI and then followed on the PI plus 3TC alone with continued suppression ^30^. These regimens are currently available and have mostly been used in the setting where there is a desire to get patients off tenofovir- and ABC-based regimens, such as those with progressive renal disease and risk factors for cardiovascular events or who are HLA-B5701 positive and therefore not good candidates for ABC. The need for this type of strategy has been somewhat attenuated by the availability of TAF.

Two large phase III studies demonstrated the activity of an NRTI and PI-sparing regimen to maintain viral suppression in those without underlying resistance. Two identically designed studies called SWORD 1 and 2 randomized patients to continue their current regimen or switch to once-daily DTG plus RPV at approved doses ^31^. The new strategy met non-inferiority criteria to the control arm and was well tolerated. In addition to being NRTI- and PI-sparing, the total mass of these pills is 75 mg, which is likely to result in a very small single-tablet regimen option.

One of the most exciting advances in treatment relates to the potential for long-acting regimens that would not require daily dosing. The strategy furthest along in clinical development is intramuscular nano-formulations of RPV and a new INSTI, cabotegravir (CAB). A pilot study, LATTE 1, demonstrated that short-acting RPV and CAB was effective in maintaining viral suppression ^32^. This was followed by LATTE 2, which was a phase II study that initially suppressed viral load with two NRTIs plus short-acting CAB, then added short-acting RPV for 4 weeks to assure it was tolerated before stopping the drug in two groups and switching to either once-monthly or once-every-two-month intragluteal injections of nano-formulations of both RPV and CAB ^33^. The study ultimately showed the same level of viral suppression in the two long-acting regimens compared to continued two NRTIs plus CAB, with results modestly favoring once-monthly over every-other-month dosing. Importantly, the novel regimen was well tolerated and found to be highly acceptable in those assigned to the regimen. A fully powered phase III study of monthly long-acting formulations of RPV plus CAB is now underway.

## Conclusion

There have been extraordinary advances in the development of ART for the treatment of HIV infection. We are now at the point where the majority of patients can be treated with a single pill per day with good tolerance and very high rates of viral suppression. New drugs in development may further expand the options available for patients. Moreover, novel regimens are in development that will allow for continued suppression with potentially fewer drugs, improved tolerability, and smaller pills without compromising efficacy. If ongoing trials are successful, we may be only a few years away from being able to offer patients treatment on a once-monthly basis.

## Abbreviations

3TC, lamivudine; ABC, abacavir; AIDS, acquired immunodeficiency syndrome; ART, antiretroviral therapy; ATV, atazanavir; CAB, cabotegravir; COBI, cobicistat; DRV, darunavir; DTG, dolutegravir; EFV, efavirenz; EVG, elvitegravir; FTC, emtricitabine; HIV, human immunodeficiency virus; INSTI, integrase strand transfer inhibitor; LPV/r, lopinavir/ritonavir; NNRTI, non-nucleoside reverse transcriptase inhibitor; NRTI, nucleoside reverse transcriptase inhibitor; PI, protease inhibitor; TAF, tenofovir alafenamide; TDF, tenofovir disoproxil fumarate; RAL, raltegravir; RPV, rilpivirine; RTV, ritonavir.
